# The Function of FoxK Transcription Factors in Diseases

**DOI:** 10.3389/fphys.2022.928625

**Published:** 2022-07-12

**Authors:** Mujun Yu, Haozhen Yu, Nan Mu, Yishi Wang, Heng Ma, Lu Yu

**Affiliations:** ^1^ School of Life Sciences, Yan’an University, Yan’an, China; ^2^ School of Basic Medical Sciences, Shaanxi University of Traditional Chinese Medicine, Xianyang, China; ^3^ Department of Physiology and Pathophysiology, School of Basic Medicine, Fourth Military Medical University, Xi’an, China; ^4^ Department of Pathology, Xijing Hospital, Fourth Military Medical University, Xi’an, China

**Keywords:** FOXK1, FOXK2, autophagy, cancer, metabolic disease

## Abstract

Forkhead box (FOX) transcription factors play a crucial role in the regulation of many diseases, being an evolutionarily conserved superfamily of transcription factors. In recent years, FoxK1/2, members of its family, has been the subject of research. Even though FoxK1 and FoxK2 have some functional overlap, increasing evidence indicates that the regulatory functions of FoxK1 and FoxK2 are not the same in various physiological and disease states. It is important to understand the biological function and mechanism of FoxK1/2 for better understanding pathogenesis of diseases, predicting prognosis, and finding new therapeutic targets. There is, however, a lack of comprehensive and systematic analysis of the similarities and differences of FoxK1/2 roles in disease, prompting us to perform a literature review.

## Introduction

Forkhead box (FOX) proteins are a family of evolutionarily conserved transcription factors characterized by highly conserved “fork-head” and “winged-helix” DNA binding domains (DBD) that specifically bind to the highly conserved sequence 5′-TTGTTTAC-3′. Based on sequence homology, members of the mammalian FOX family can be further divided into 19 subfamilies, namely FoxA to FoxS (Lam et al., 2013). Their role in regulating the expression of target genes contributes to various cellular functions, including cell cycle, cell growth, proliferation, differentiation, programmed death, metabolism, DNA damage, drug resistance, angiogenesis, and carcinogenesis (Huang and Lee 2004).

FoxK (FoxK1 and FoxK2 collectively known as FoxK) are members of the Forkhead transcriptional family, which are expressed in all tissues and organs and play an essential role. FoxK is highly conserved in structure and function. It regulates cell proliferation, survival, skeletal muscle regeneration, myogenic differentiation and various tumors genesis and development through transcriptional regulation (Katoh and Katoh 2004). It has been identified that FoxK1/2 gene structure and epigenetic abnormalities are closely associated with the development of various human diseases, but the detailed biological mechanisms are not yet known. Further exploration of the function of FoxK1/2 protein will help to reveal the pathogenesis of related diseases and explore preventive and therapeutic measures. The present paper describes FoxK1/2 gene and protein structure, biological function, and analyzes the role of FoxK1/2 in different diseases.

## Structure and Function of FOXK1/2

### Biological Structure

**FIGURE 1:**
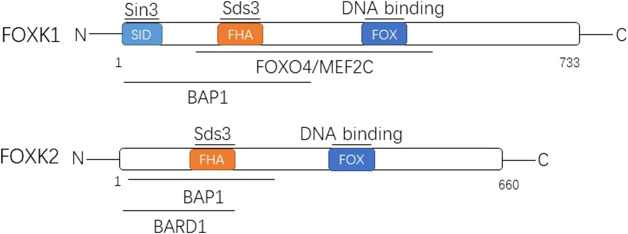
The two members of FOXK family are shown with their domain arrangement. Letters within the bar indicate structural domains. Some of the well-known proteins interacting with FOXK1 and FOXK2 are shown above the lines at the corresponding domains. FOX, forkhead winged helix-turn-helix DNA binding domain; FHA, forkhead-associated domain; SID, Sin3b-interacting domain; Sds3, suppressor of defective silencing 3; FOXO4/MEF2C, Forkhead Box Class O4/Myocyte Enhancer Factor-2C; BAP1, BRCA associated protein 1; BARD1, BRCA1 associated ring domain 1.

FoxK1 and FoxK2 are structurally similar, and both have two domains. One is the FOX domain, containing the DNA domain, which mainly recognizes the promoter region containing the 5′-GTAACA-3′ standard sequence (He et al., 2018; Sakaguchi et al., 2019) and can bind directly to DNA. One is the forhkead-associated domain (FHA), a protein interaction domain that mainly mediates the binding of FoxK to protein phosphorylation residues (Liu et al., 2019). Both domains are highly conserved in FoxK1 and FoxK2, mediating their interactions with other proteins and regulating cell cycle dynamics (Figure 1).

The encoding gene of FoxK1 is located on chromosome 7p22.1 and encodes a total of 733 amino acids that produce the FoxK1 protein (Huang and Lee 2004). There are two distinct subtypes, namely FoxK1a and FoxK1b. FoxK1, also known originally as myocyte nuclear factor, is based on a restricted expression pattern in the myogenic lineage of mouse embryogenesis and was discovered in Williams’ laboratory (Bassel-Duby et al., 1994). The FHA domain of FOXK1 can interact with defective silencing inhibitor 3 (Sds3) (Shi et al., 2012a). Moreover, the n-terminal contains SID (a 3-interaction domain independent of Swi) and can interact directly with Sin3 (Shi and Garry 2012). It also interacts with FoxO4 (Forkhead Box Class O4), MEF2C(Myocyte Enhancer Factor-2C), and BAP1 (BRCA-associated protein 1) (Shi et al., 2012b; Okino et al., 2015).

The encoding gene of FoxK2 is located on chromosome 17q25.3 and encodes 660 amino acids, producing the corresponding FoxK2 protein (Huang and Lee 2004). It has three different subtypes. The FHA domain of FoxK2 can also interact with Sds (Wang et al., 2015). Moreover, its N-terminal is related to the BAP1 interaction (Okino et al., 2015) and can interact with BARD1 (BRCA1-related ring domain 1) as well (Liu et al., 2015), which is similar to FoxK1.

FoxK1/2 is an essential transcription factor that regulates cell proliferation, survival, skeletal muscle regeneration, myogenic differentiation, and cancer development (Katoh and Katoh 2004). Previous studies have shown that FoxK1/2 is closely associated with various diseases and plays a complex role in tumor therapy especially.

### Biological Function

#### Regulated Target Genes

FoxK1/2 plays an irreplaceable role in many diseases as an essential transcription factor. FoxK1 overexpression can eliminate the inhibitory effect of Mir-137 and activate the Wnt/β-catenin pathway to promote cell growth (Ji, Jiang, and Zhang 2018). Ectopic expression of FoxK1 can up-regulate its target genes (zinc finger E-box binding homeobox 1), AP-1, and TERT (telomerase reverse transcriptase). FoxK1 also promotes epithelial-mesenchymal transition (EMT) of glioblastoma pleomorphic cells by activating transcription of its downstream target gene, Snail (Xu et al., 2018). However, FoxK1 inhibits the EMT process by suppressing the expression of its target gene Twis (inducer of EMT) in breast cancer cell line McF-7 (Sun et al., 2016). The miRNA-646 has been shown to target FoxK1 and inhibit its function in promoting proliferation and EMT-induced metastasis (Zhang et al., 2017). FHL2 (four and a half LIM domains 2) is a LIM protein that interacts directly with FoxK1 through its LIM domain to form a complex and inhibit FoxO4 transcriptional activity in myogenic progenitor cells (Shi, Bowlin, and Garry 2010). During mitosis, FoxK1 can act as a transcriptional repressor of p21, interacting with scaffold protein, JNK-associated leucine zipper protein (JLP), and PLK1 (polo like kinase 1) to regulate cell cycle-dependent gene expression (Ramkumar et al., 2015).

For FoxK2, the EMT process of McF-7 cells can be inhibited mainly by repressing Ezh2 transcription, and the EMT progression of these cells can also be inhibited by repressing target genes such as N-cadherin and Snail (Chen et al., 2017; Wang et al., 2018).

Since several ncRNAs directly regulate FoxK protein (Lin et al., 2017; Zhang et al., 2017; Ma et al., 2018), the targeted therapeutic regulation of FoxK on ncRNA may also be a valuable therapeutic strategy.

#### FoxK1/2 and Autophagy

Autophagy is a well-preserved eukaryotic catabolic process that promotes homeostasis and ensures cell survival. During stress, such as starvation, cells generate membrane-bound autophagosomes that engulf cytoplasmic proteins, lipids, and organelles (Mizushima 2007). These substances are then transported to the lysosome for degradation, which contributes to the cellular reorganization during tissue development and differentiation and the production of metabolites necessary to maintain energy requirements under nutrient constraints. Autophagy has been considered a cytoplasmic phenomenon regulated solely by cytoplasmic complexes. However, a growing number of studies have shown that autophagy is sensitive to epigenetic and transcriptional changes (Bowman, Ayer, and Dynlacht 2014).

Related studies show that DNA damage mediated FoxK cytoplasmic capture induces autophagy because FoxK (FoxK1 and FxoK2) can act as transcriptional inhibitors of autophagy-related genes (ATGs). A cancer derived FoxK mutation induces FoxK hyperphosphorylation and enhances autophagy, leading to resistance to chemotherapy. Combination therapy with cisplatin and chloroquine can overcome chemotherapy resistance caused by FoxK mutation (Chen et al., 2020).

It is also observed that FoxK1/2 counteracts the effect of another Foxo3 transcription factor that induces an overlapping set of autophagy and atrophy targets in muscle. FoxK1/2 specifically recruits the SIN3a-HDAC complex to inhibit the acetylation of histone H4 and the expression of key autophagy genes (Bowman, Ayer, and Dynlacht 2014).

#### FOXK1/2 and Ubiquitination

According to relevant studies, FoxK2 can bind SIN3A and PR-DUB complexes. The PR-DUB complex contains a vital tumor suppressor protein, the deubiquitin enzyme BAP1. FoxK2 recruits BAP1 into DNA (Okino et al., 2015) and promotes local histone deubiquitination, leading to changes in target gene activity (Ji et al., 2014).

ASXL1 (ASXL transcriptional regulator 1) interacts with the fork head transcription factors FoxK1 and FoxK2 to regulate a subset of FoxK1/2 target genes. It was found that the mutant ASXL1 protein with c-terminal truncation was expressed at a much higher level in ASXL1 heterozygous leukemia cells than the wild-type protein and lost its ability to interact with FoxK1/2. Specific deletion of the mutant allele eliminates Ce-terminal truncated ASXL1 expression and increases the association between wild-type ASXL1 and BAP1, thereby restoring the expression of the bap1-ASXL1-FoxK1/2 target gene (Xia et al., 2021).

**FIGURE 2:**
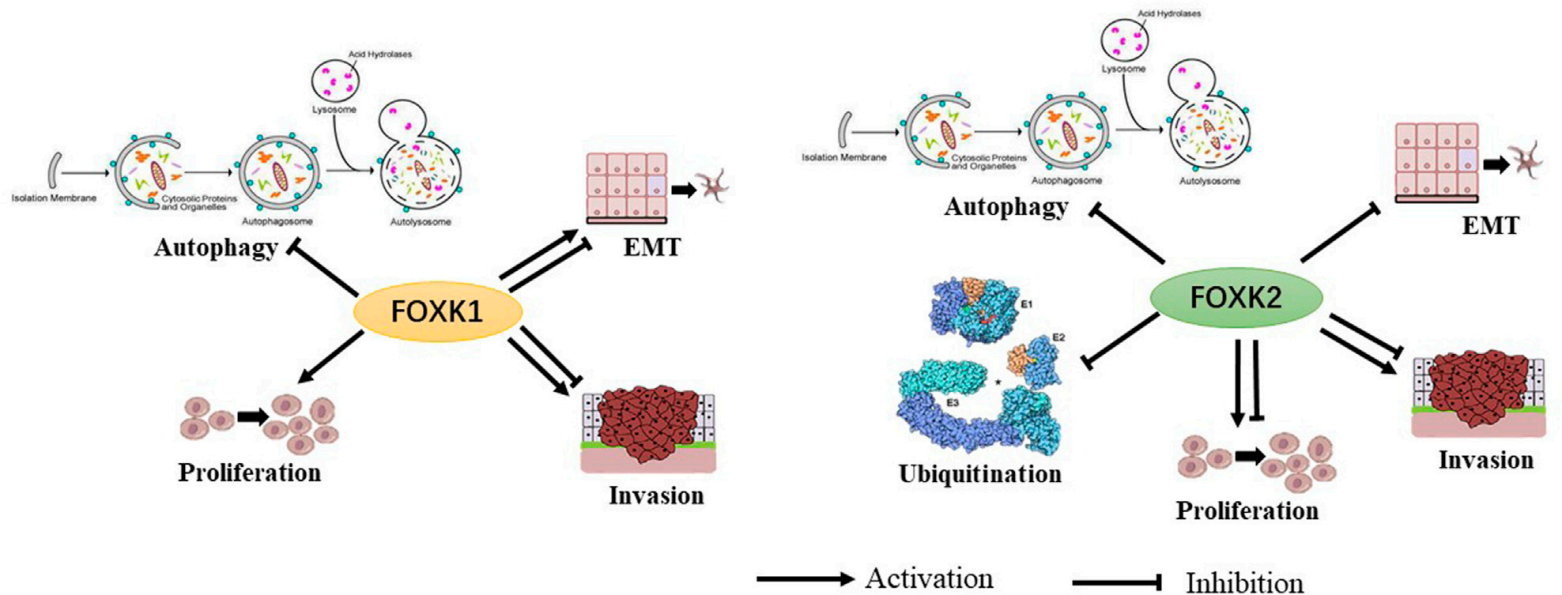
The biological functions of FoxK1 and FoxK2 have been shown to have multiple permissive and inhibitory effects.

These studies demonstrate the biological role of FoxK1/2 as transcription factors. However, the function and mechanism of FoxK1/2 in disease and progression remains largely unknown (Figure 2).

## The Role of FoxK1/2 in Disease

### FoxK1/2 and Cancer

#### Breast Cancer

In breast cancer, FoxK1 has both stimulatory and inhibitory effects, while FoxK2 has inhibitory effects. The average expression of Foxk1 gene has been reported to be lower in breast cancer tissues than in its adjacent tissues, and patients with low FoxK1 expression have a worse prognosis effect than with high expression. Endothelial tube formation assays indicated that Foxk1 might regulate breast cancer angiogenesis through transcriptional repression of vascular endothelial growth factor (Sun et al., 2016). However, other studies concluded conversely that FoxK1 expression was significantly higher in breast cancer tissues than in adjacent tissues through QRT-PCR detection. The Patients with high FoxK1 expression had significantly higher pathological grades than patients with low FoxK1 expression. Meanwhile, the overall survival rate was significantly lower with high expression. Its high expression was significantly correlated with tumor TNM stage, tissue grade and lymph node metastasis.

FoxK2 can inhibit the proliferation and invasion of breast cancer cells and suppress the growth and metastasis of breast cancer. FoxK2 is activated by ERα trans, and the transcriptional inhibition occurs through continuous feedback from HIF1β (hypoxia-inducible factor 1β)/EZH2 (enhancer of zeste 2 polycomb repressive complex 2 subunit) (Nestal de Moraes et al., 2015). Studies have shown that FOXK2 can inhibit the proliferation and metastasis of breast cancer *in vivo* and *in vitro*. Mechanistically, FOXK2 inhibits the transcription of a series of oncogenes, including HIF1β, EZH2 and VEGFR, by binding to SIN3A, NCoR (nuclear receptor corepressor 1)/SMRT (nuclear receptor corepressor 2), NuRD and other transcription inhibition complexes. It affects a series of signaling pathways, including the cell cycle, DNA damage response, p53, hypoxia response, EMT *etc*., thereby inhibiting the occurrence and development of breast cancer (Shan et al., 2016).

Conversely, FoxK2 plays a central role in the cytotoxic drug responses in breast cancer. Cloning and cell viability analysis showed that enhanced FoxK2 expression sensitized McF-7 breast cancer cells to paclitaxel or epirubicin treatment while depletion of FoxK2 through small interfering RNA (siRNA) resulted in resistance. Previously data also showed that the activation of tumor suppressor FoxO3a by paclitaxel and epirubicin is mediated by FoxK2 induction (Peng et al., 2016).

#### Gastric Cancer

Interestingly, FoxK1/2 play different roles in gastric cancer, FoxK1 promoting and FoxK2 inhibiting it. FoxK1 protein expression is higher in 80% of fresh cancer tissues than in adjacent normal tissues. FoxK1 overexpression enhances gastric cancer cells proliferation, migration, and invasion. In addition, transforming growth factor -β1 (TGF-β1) stimulates FoxK1 expression (Zhang et al., 2019). FoxK1 can physically interact with and stabilize vimentin, and FoxK1 positively correlates with vimentin expression in gastric cancer cells. Elevated expression levels of both proteins were significantly associated with differentiation, lymph node metastasis, staging, and poor prognosis of AJCC’s current staging system. In addition, the co-expression of FoxK1 and vimentin promotes the metastasis of gastric cancer cells by inducing EMT (Zhang et al., 2019).

FoxK2 has an inhibitory effect on gastric cancer. High-grade gastric cancer tissues express low levels of FoxK2 (Liu et al., 2018). The results showed that high FoxK2 expression predicted a better prognosis, indicating that FoxK2 may be a therapeutic target for gastric cancer and a prognostic milestone for patients with different stages of gastric cancer. In addition, the results showed that FoxK2 overexpression reduced cell invasion, growth, and proliferation. Generally, FoxK2 inhibits the progression of the cell cycle, which plays an important role in how cells change and adapt throughout the cycle (Qian, Xia, and Feng 2017).

#### Colorectal Cancer

Colorectal cancer is influenced by both FoxK1 and FoxK2, with they all promoting the tumor. FoxK1 is highly expressed in colorectal cancer cells and tissues. It is suggested that the interaction between FoxK1 and FHL2 promotes the growth and metastasis of colorectal cancer cells. FoxK1 is a cell cycle and growth regulator that inhibits apoptosis in colon cancer cells. Downregulation of the FoxK1 gene can induce G0/G1 cell cycle arrest in colorectal cancer cells, inhibit the growth of colorectal cancer cells, promote apoptosis, and increase cell sensitivity to 5-fluorouracil (5-FU) induced apoptosis (Wu et al., 2016). In addition, experimental results have also shown that up-regulation of FoxK1 can reverse the inhibitory effect of miR-497-5p on proliferation, anti-apoptotic activity, and metastasis of colorectal cancer cells (Wang et al., 2020).

FoxK2 is significantly upregulated in human colorectal cancer tissues, which correlates with aggressive characteristics and suggests a poor prognosis. Overexpression of FoxK2 promotes migration, invasion, and metastasis of colorectal cancer. There is more FOXK2 expression in colorectal cancer tissues than in normal tissues, and in metastatic tissues than in primary tissues, and in primary tissues of metastatic patients than in non-metastatic patients, which is associated with poor prognosis. Mechanistically, FOXK2 can be involved in hepato-lung metastasis of colorectal cancer through transcription activation of epidermal growth factor receptor (EGFR), and conversely, the activated EGFR signaling pathway can also activate FOXK2 through extracellular signal-regulated protein kinase (ERK) and nuclear factor κB (NF-κB). FOXK2 can also transcriptionally activate EMT and drive transcription factor ZEB1 to promote EMT and invasion and metastasis of colorectal cancer (Du et al., 2019). Studies have shown that oncogene SOX9 regulates FoxK2 upregulation by binding directly to the FoxK2 promoter, and the loss of FoxK2 attenuated SOX9-induced cell growth. Furthermore, FoxK2 expression correlated with SOX9 expression significantly (Cui et al., 2018).

#### Liver Cancer

Studies have shown that FoxK1 was upregulated in hepatocellular carcinoma cells compared to normal liver cells. FoxK1 downregulation reduced cell viability and HK2 expression. FoxK1 gene knockout reduced glucose consumption and lactic acid production in HCC cells. In addition, FoxK1 downregulation inhibited Akt/mTOR pathway activation. Inhibition of the Akt/mTOR pathway reduced viability and glycolysis in hepatocellular carcinoma cells (Kong et al., 2020).

FoxK2 functions as the oncogene and downregulation of FoxK2 can inhibit proliferation, colony formation, migration, and invasion in Hep3B and LM3 cells (Khemlina, Ikeda, and Kurzrock 2017). FoxK2 is significantly elevated in HCC cells and correlates to tumor size, TNM stage, and tumor vascular infiltration. Up-regulation of FoxK2 in HCC cells advances cell proliferation and migration (Shan et al., 2016). FoxK2 may be involved in developing liver cancer, and affect the initiation of tumor promoters by inhibiting cell proliferation and migration. FoxK2 can block tumor progression through the p53 pathway, hypoxia pathway, or β-catenin signaling pathway (Wang et al., 2015). In addition, FoxK2 can also induce carcinogenic activity in HCC through the PI3K/Akt signaling pathway.

In addition to breast cancer, gastric cancer, colorectal cancer, and liver cancer mentioned above, FoxK1/2 can also regulate other tumor cells, cellular homeostasis, and biological behavior through different mechanisms.

Overall, Foxk1/2 has dual functions in tumors, acting as either an oncogene or a tumor suppressor (Table 1). Although FOXK1 and FOXK2 have shown partial functional duplication in different studies, their regulatory roles in tumors are not completely equivalent.

**TABLE 1:** Functional roles of FOXKs pathway in different types of cancer.

Cancer types	Members of FOXK family	Cell lines	Key message(s)
Breast Cancer	FoxK1	Cell lines MCF-7 and MDA-MB-231 cells	High FOXK1 expression is associated with better prognosis. FOXK1 regulates breast cancer angiogenesis through inhibition of vascular endothelial growth factor
	FoxK2	Cell lines MCF-7	FoxK2 is activated by ERα trans, and the transcriptional inhibition occurs through continuous feedback from HIF1β/EZH2
Gastric Cancer	FoxK1	the human gastric cancer (GC) cell lines BCG823 and SGC7901	The co-expression of FoxK1 and vimentin promotes the metastasis of gastric cancer cells by inducing EMT. Functional roles of FOXKs pathway in different types of cancer
	FoxK2	HCT116, SW480, SW620, DLD-1, LOVO, COLO205 and HEK293T cell line	FoxK2 overexpression reduced cell invasion, growth, and proliferation. FoxK2 inhibits the progression of the cell cycle
Colorectal Cancer	FoxK1	CRC cells	FoxK1 and FHL2 promotes the growth and metastasis of colorectal cancer cells
	FoxK2	CRC cells	FOXK2 can be involved in hepato-lung metastasis of colorectal cancer through transcription activation of epidermal growth factor receptor (EGFR)
			Oncogene SOX9 regulates FoxK2 upregulation by binding directly to the FoxK2 promoter, and the loss of FoxK2 attenuated SOX9-induced cell growth
Liver Cancer	FoxK1	HCC cells	FoxK1 downregulation inhibited Akt/mTOR pathway activation. Inhibition of the Akt/mTOR pathway reduced viability and glycolysis in hepatocellular carcinoma cells
	FoxK2	HCC cells	Up-regulation of FoxK2 in HCC cells advances cell proliferation and migration

### FoxK1/2 and Metabolic Disease

At the transcriptional level, FoxK1/2 can directly induce the expression of glycolytic-related kinases and pyruvate dehydrogenase kinases such as hexokinase, phosphofructokinase, pyruvate kinase, and lactate dehydrogenase to maintain aerobic glycolysis and inhibit mitochondrial oxidation (Sukonina et al., 2019).

FoxK1 is a mediator of gene expression regulation by mTORC1. mTORC1 inhibits glycogen synthase activator 3 (Gsk3), which mediates an increase in FOXK1 phosphorylation, resulting in a decrease in DNA binding and nuclear rejection. FOXK1 can also regulate glucose, serine, and nucleotide metabolism directly or by inducing the hypoxia-inducible factor 1α (HIF1α) transcription factor in response to the mTORC1 signaling pathway (He et al., 2018). *In vitro* and *in vivo* experiments, including studies on primitive human cells, suggested that FoxK1 and FoxK2 may be essential regulators of reprogramming cellular metabolism to induce aerobic glycolysis.

FoxK1 and FoxK2 also act as downstream signaling molecules of Akt in insulin signaling. FoxK1 and FoxK2 are phosphorylated by the Akt/mTOR pathway following insulin stimulation, resulting in their translocation to the nucleus, controlling gene expression associated with mitochondrial metabolism and cell proliferation (Sakaguchi et al., 2019). Interestingly, one study showed that weight loss led to a decrease in NNMT expression, a decrease in FoxK2, and a parallel increase in FBX021 expression. This NNMT-induced regulation may be mediated by epigenetic regulation, with the DNA methylation level of FoxK2 inversely paralleling its gene expression (Crujeiras et al., 2018).

SOX9 binds directly to the FoxK2 promoter and regulates the up-regulation of FoxK2. Fibroblast-specific loss of SOX9 has been reported to improve left ventricular dysfunction, dilation, and cardiac scarring induced by MI in the heart. Fibroblast SOX9 is a primary regulator of cardiac fibrosis and inflammation (Scharf et al., 2019). Cardiomyocyte-specific SOX9 mediates hypertrophy and early fibrosis following cardiac pressure overload. However, SOX9 deficiency delays cardiac growth and remodeling and fails to maintain cardiac function. Studies have shown that SOX9 driven by cardiac myocytes initiates a hypertrophic cascade reaction, which may involve crosstalk between myocytes and fibroblasts (Schauer et al., 2019). Therefore, it is worth evaluating and exploring whether SOX9 can regulate cardiac fibrosis or any other cardiomyopathy through FoxK1/2.

## Research Status and Prospects

Members of the FoxK family regulate the expression of a variety of genes during cell proliferation, apoptosis, cell cycle progression, DNA damage, and carcinogenesis and are essential regulators of various physiological and pathological processes. FoxK1/2 regulates the expression of a series of downstream target genes as a transcription factor, thereby affecting multiple signaling pathways and biological behaviors of cancer cells. The research on FoxK1/2 is still at the introductory stage, and more and more clinical data are needed to support its mechanism, expression, prognostic effects, and clinical applications in different tumors. Therefore, as the mechanism of FoxK1/2 action is further elucidated in future studies, the discovery of more FoxK1/2 regulatory targets will be of great significance to reveal the pathogenesis and prognosis of tumors and other diseases. In addition, the role of FoxK1/2 in non-neoplastic diseases, such as cardiovascular diseases and metabolic diseases, also deserves attention.

Future research on drugs targeting FoxK1/2-related regulatory pathways will be more challenging, requiring multidisciplinary collaboration among pharmacy, basic medicine, and clinical science.

## Abbreviations

ATGs, autophagy-related genes; BAP1, BRCA-associated protein 1; BARD1, BRCA1-related ring domain 1; DBD, DNA binding domains; EGFR, epidermal growth factor receptor; EMT, epithelial-mesenchymal transition; ERK, signal-regulated protein kinase; FHA, forkhead-associated domain; FHL2, four and a half LIM domains 2; FOX proteins, Forkhead box proteins; FoxO4, Forkhead Box Class O4; Gsk3, glycogen synthase activator 3; HIF1α, hypoxia-inducible factor 1α; MEF2C, Myocyte Enhancer Factor-2C; NF-κB, nuclear factor κB; PLK1, polo like kinase 1; Sds3, suppressor of defective silencing 3; SID, Sin3b-interacting domain; TERT, telomerase reverse transcriptase; ZEB1, zinc finger E-box binding homeobox 1.

## References

[B1] Bassel-Duby R. Hernandez M. D. Yang Q. Rochelle J. M. Seldin M. F. Williams R. S. (1994). Myocyte Nuclear Factor, a Novel Winged-Helix Transcription Factor under Both Developmental and Neural Regulation in Striated Myocytes. Mol. Cell. Biol. 14, 4596–4605. 10.1128/mcb.14.7.4596 8007964 PMC358832

[B2] Bowman C. J. Ayer D. E. Dynlacht B. D. (2014). Foxk Proteins Repress the Initiation of Starvation-Induced Atrophy and Autophagy Programs. Nat. Cell Biol. 16, 1202–1214. 10.1038/ncb3062 25402684 PMC4250422

[B3] Chen S. Jiang S. Hu F. Xu Y. Wang T. Mei Q. (2017). Foxk2 Inhibits Non-small Cell Lung Cancer Epithelial-Mesenchymal Transition and Proliferation through the Repression of Different Key Target Genes. Oncol. Rep. 37, 2335–2347. 10.3892/or.2017.5461 28260088

[B4] Chen Y. Wu J. Liang G. Geng G. Zhao F. Yin P. (2020). CHK2-FOXK axis Promotes Transcriptional Control of Autophagy Programs. Sci. Adv. 6, eaax5819. 10.1126/sciadv.aax5819 31911943 PMC6938702

[B5] Crujeiras A. B. Pissios P. Moreno-Navarrete J. M. Diaz-Lagares A. Sandoval J. Gomez A. (2018). An Epigenetic Signature in Adipose Tissue Is Linked to Nicotinamide N-Methyltransferase Gene Expression. Mol. Nutr. Food Res. 62, e1700933. 10.1002/mnfr.201700933 29688621

[B6] Cui H. Gao Q. Zhang L. Han F. Wang L. (2018). Knockdown of FOXK1 Suppresses Liver Cancer Cell Viability by Inhibiting Glycolysis. Life Sci. 213, 66–73. 10.1016/j.lfs.2018.10.018 30312701

[B7] de Moraes G. N. Khongkow P. Gong C. Yao S. Gomes A. R. Ji Z. (2015). Forkhead Box K2 Modulates Epirubicin and Paclitaxel Sensitivity through FOXO3a in Breast Cancer. Oncogenesis 4, e167. 10.1038/oncsis.2015.26 26344694 PMC4767938

[B8] Du F. Qiao C. Li X. Chen Z. Liu H. Wu S. (2019). Forkhead Box K2 Promotes Human Colorectal Cancer Metastasis by Upregulating ZEB1 and EGFR. Theranostics 9, 3879–3902. 10.7150/thno.31716 31281520 PMC6587343

[B9] He L. Gomes A. P. Wang X. Yoon S. O. Lee G. Nagiec M. J. (2018). mTORC1 Promotes Metabolic Reprogramming by the Suppression of GSK3-dependent Foxk1 Phosphorylation. Mol. Cell 70, 949–960. 10.1016/j.molcel.2018.04.024 29861159 PMC6591025

[B10] Huang J. Lee V. (2004). Identification and Characterization of a Novel Human FOXK1 Gene In Silico. Int. J. Oncol. 25, 751–757. 10.3892/ijo.25.3.751 15289879

[B11] Ji Z. G. Jiang H. T. Zhang P. S. (2018). FOXK1 Promotes Cell Growth through Activating Wnt/β-Catenin Pathway and Emerges as a Novel Target of miR-137 in Glioma. Am. J. Transl. Res. 10, 1784–1792. https://www.ncbi.nlm.nih.gov/pmc/articles/PMC6038085/ 30018719 PMC6038085

[B12] Ji Z. Mohammed H. Webber A. Ridsdale J. Han N. Carroll J. S. (2014). The Forkhead Transcription Factor FOXK2 Acts as a Chromatin Targeting Factor for the BAP1-Containing Histone Deubiquitinase Complex. Nucleic Acids Res. 42, 6232–6242. 10.1093/nar/gku274 24748658 PMC4041447

[B13] Katoh M. Katoh M. (2004). Identification and Characterization of Human FOXK1 Gene In Silico. Int. J. Mol. Med. 14, 127–132. 10.3892/ijmm.14.1.127 15202027

[B14] Khemlina G. Ikeda S. Kurzrock R. (2017). The Biology of Hepatocellular Carcinoma: Implications for Genomic and Immune Therapies. Mol. Cancer 16, 149. 10.1186/s12943-017-0712-x 28854942 PMC5577674

[B15] Kong J. Zhang Q. Liang X. Sun W. (2020). FOXK2 Downregulation Suppresses EMT in Hepatocellular Carcinoma. Open Med. (Wars) 15, 702–708. 10.1515/med-2020-0129 33313412 PMC7706124

[B16] Lam E. W.-F. Brosens J. J. Gomes A. R. Koo C.-Y. (2013). Forkhead Box Proteins: Tuning Forks for Transcriptional Harmony. Nat. Rev. Cancer 13, 482–495. 10.1038/nrc3539 23792361

[B17] Lin M.-F. Yang Y.-F. Peng Z.-P. Zhang M.-F. Liang J.-Y. Chen W. (2017). FOXK2, Regulted by miR-1271-5p, Promotes Cell Growth and Indicates Unfavorable Prognosis in Hepatocellular Carcinoma. Int. J. Biochem. Cell Biol. 88, 155–161. 10.1016/j.biocel.2017.05.019 28506857

[B18] Liu X. Wei X. Niu W. Wang D. Wang B. Zhuang H. (2018). Downregulation of FOXK2 Is Associated with Poor Prognosis in Patients with Gastric Cancer. Mol. Med. Rep. 18, 4356–4364. 10.3892/mmr.2018.9466 30221666 PMC6172389

[B19] Liu Y. Ao X. Jia Z. Bai X.-Y. Xu Z. Hu G. (2015). FOXK2 Transcription Factor Suppresses ERα-Positive Breast Cancer Cell Growth Through Down-Regulating the Stability of ERα via Mechanism Involving BRCA1/BARD1. Sci. Rep. 5, 8796. 10.1038/srep08796 25740706 PMC4350111

[B20] Liu Y. Ding W. Ge H. Ponnusamy M. Wang Q. Hao X. (2019). FOXK Transcription Factors: Regulation and Critical Role in Cancer. Cancer Lett. 458, 1–12. 10.1016/j.canlet.2019.05.030 31132431

[B21] Ma X. Yang X. Bao W. Li S. Liang S. Sun Y. (2018). Circular RNA circMAN2B2 Facilitates Lung Cancer Cell Proliferation and Invasion via miR-1275/FOXK1 axis. Biochem. Biophysical Res. Commun. 498, 1009–1015. 10.1016/j.bbrc.2018.03.105 29550475

[B22] Mizushima N. (2007). Autophagy: Process and Function. Genes Dev. 21, 2861–2873. 10.1101/gad.1599207 18006683

[B23] Okino Y. Machida Y. Frankland-Searby S. Machida Y. J. (2015). BRCA1-associated Protein 1 (BAP1) Deubiquitinase Antagonizes the Ubiquitin-Mediated Activation of FoxK2 Target Genes. J. Biol. Chem. 290, 1580–1591. 10.1074/jbc.m114.609834 25451922 PMC4340404

[B24] Peng Y. Zhang P. Huang X. Yan Q. Wu M. Xie R. (2016). Direct Regulation of FOXK1 by C-Jun Promotes Proliferation, Invasion and Metastasis in Gastric Cancer Cells. Cell Death Dis. 7, e2480. 10.1038/cddis.2016.225 27882939 PMC5260906

[B25] Qian Y. Xia S. Feng Z. (2017). Sox9 Mediated Transcriptional Activation of FOXK2 Is Critical for Colorectal Cancer Cells Proliferation. Biochem. Biophysical Res. Commun. 483, 475–481. 10.1016/j.bbrc.2016.12.119 28007600

[B26] Ramkumar P. Lee C. M. Moradian A. Sweredoski M. J. Hess S. Sharrocks A. D. (2015). JNK-Associated Leucine Zipper Protein Functions as a Docking Platform for Polo-like Kinase 1 and Regulation of the Associating Transcription Factor Forkhead Box Protein K1. J. Biol. Chem. 290, 29617–29628. 10.1074/jbc.m115.664649 26468278 PMC4705960

[B27] Sakaguchi M. Cai W. Wang C.-H. Cederquist C. T. Damasio M. Homan E. P. (2019). FoxK1 and FoxK2 in Insulin Regulation of Cellular and Mitochondrial Metabolism. Nat. Commun. 10, 1582. 10.1038/s41467-019-09418-0 30952843 PMC6450906

[B28] Scharf G. M. Kilian K. Cordero J. Wang Y. Grund A. Hofmann M. (2019). Inactivation of Sox9 in Fibroblasts Reduces Cardiac Fibrosis and Inflammation. JCI Insight 5, e126721. 10.1172/jci.insight.126721 PMC6693887 31310588

[B29] Schauer A. Adams V. Poitz D. M. Barthel P. Joachim D. Friedrich J. (2019). Loss of Sox9 in Cardiomyocytes Delays the Onset of Cardiac Hypertrophy and Fibrosis. Int. J. Cardiol. 282, 68–75. 10.1016/j.ijcard.2019.01.078 30765281

[B30] Shan L. Zhou X. Liu X. Wang Y. Su D. Hou Y. (2016). FOXK2 Elicits Massive Transcription Repression and Suppresses the Hypoxic Response and Breast Cancer Carcinogenesis. Cancer Cell 30, 708–722. 10.1016/j.ccell.2016.09.010 27773593

[B31] Shi X. Bowlin K. M. Garry D. J. (2010). Fhl2 Interacts with Foxk1 and Corepresses Foxo4 Activity in Myogenic Progenitors. Stem Cells 28, 462–469. 10.1002/stem.274 20013826

[B32] Shi X. Garry D. J. (2012). Sin3 Interacts with Foxk1 and Regulates Myogenic Progenitors. Mol. Cell Biochem. 366, 251–258. 10.1007/s11010-012-1302-2 22476904 PMC4494651

[B33] Shi X. Wallis A. M. Gerard R. D. Voelker K. A. Grange R. W. DePinho R. A. (2012b). 'Foxk1 Promotes Cell Proliferation and Represses Myogenic Differentiation by Regulating Foxo4 and Mef2 Factors. J. Cell Sci. 125, 5329–5337. 10.1242/jcs.105239 22956541 PMC3561855

[B34] Shi X. Seldin D. C. Garry D. J. (2012a). Foxk1 Recruits the Sds3 Complex and Represses Gene Expression in Myogenic Progenitors. Biochem. J. 446, 349–357. 10.1042/bj20120563 22716292 PMC4494662

[B35] Sukonina V. Ma H. Zhang W. Bartesaghi S. Subhash S. Heglind M. (2019). FOXK1 and FOXK2 Regulate Aerobic Glycolysis. Nature 566, 279–283. 10.1038/s41586-019-0900-5 30700909

[B36] Sun T. Wang H. Li Q. Qian Z. Shen C. (2016). Retracted: Forkhead Box Protein K1 Recruits TET1 to Act as a Tumor Suppressor and Is Associated with MRI Detection. Jpn. J. Clin. Oncol. 46, 209–221. 10.1093/jjco/hyv185 26732382

[B37] Wang B. Zhang X. Wang W. Zhu Z. Tang F. Wang D. (2018). Forkhead Box K2 Inhibits the Proliferation, Migration, and Invasion of Human Glioma Cells and Predicts a Favorable Prognosis. Ott 11, 1067–1075. 10.2147/ott.s157126 PMC5833792 29520156

[B38] Wang N. He Jia-X. Jia Guo-Z. Wang K. Zhou S. Wu T. (2020). RETRACTED ARTICLE: The lncRNA XIST Promotes Colorectal Cancer Cell Growth through Regulating the miR-497-5p/FOXK1 axis. Cancer Cell Int. 20, 553. 10.1186/s12935-020-01647-4 33298041 PMC7727145

[B39] Wang W. Li X. Lee M. Jun S. Aziz K. E. Feng L. (2015). FOXKs Promote Wnt/β-Catenin Signaling by Translocating DVL into the Nucleus. Dev. Cell 32, 707–718. 10.1016/j.devcel.2015.01.031 25805136 PMC4374128

[B40] Wu Y. Xie R. Liu X. Wang J. Peng Y. Tang W. (2016). Knockdown of FOXK1 Alone or in Combination with Apoptosis-Inducing 5-FU Inhibits Cell Growth in Colorectal Cancer. Oncol. Rep. 36, 2151–2159. 10.3892/or.2016.5041 27571921

[B41] Xia Y.-K. Zeng Y.-R. Zhang M.-L. Liu P. Liu F. Zhang H. (2021). Tumor-derived Neomorphic Mutations in ASXL1 Impairs the BAP1-ASXL1-FOXK1/K2 Transcription Network. Protein Cell 12, 557–577. 10.1007/s13238-020-00754-2 32683582 PMC8225741

[B42] Xu H. Huang S. Zhu X. Zhang W. Zhang X. (2018). FOXK1 Promotes Glioblastoma Proliferation and Metastasis through Activation of Snail Transcription. Exp. Ther. Med. 15, 3108–3116. 10.3892/etm.2018.5732 29456714 PMC5795754

[B43] Zhang H. Wu X. Xiao Y. Wu L. Peng Y. Tang W. (2019). Coexpression of FOXK1 and Vimentin Promotes EMT, Migration, and Invasion in Gastric Cancer Cells. J. Mol. Med. 97, 163–176. 10.1007/s00109-018-1720-z 30483822

[B44] Zhang P. Tang W. M. Zhang H. Li Y. Q. Peng Y. Wang J. (2017). MiR-646 Inhibited Cell Proliferation and EMT-Induced Metastasis by Targeting FOXK1 in Gastric Cancer. Br. J. Cancer 117, 525–534. 10.1038/bjc.2017.181 28632723 PMC5558677

